# How Chain Intermixing Dictates the Polymorphism of PVDF in Poly(vinylidene fluoride)/Polymethylmethacrylate Binary System during Recrystallization: A Comparative Study on Core–Shell Particles and Latex Blend

**DOI:** 10.3390/polym9090448

**Published:** 2017-09-14

**Authors:** Yue Li, Guoqiang Zhang, Shaofeng Song, Haijun Xu, Mingwang Pan, Gan-Ji Zhong

**Affiliations:** 1 Institute of Polymer Science and Engineering, Hebei University of Technology, Tianjin 300130, China; liyue1990liyue@gmail.com (Y.L.); sfsongcn@163.com (S.S.); haijunluzhan@126.com (H.X.); 2 College of Polymer Science and Engineering, Sichuan University, Chengdu 610065, Sichuan, China; 3 Department of Macromolecular Science and Engineering, Case Western Reserve University, Cleveland, OH 44106-7202, USA; gxz78@case.edu

**Keywords:** PVDF/PMMA blend, core–shell nanoparticles, miscibility, polymorphism, seeded emulsion polymerization

## Abstract

In the past few decades, Poly(vinylidene fluoride)/Polymethylmethacrylate (PVDF/PMMA) binary blend has attracted substantial attention in the scientific community due to possible intriguing mechanical, optical and ferroelectric properties that are closely related to its multiple crystal structures/phases. However, the effect of PMMA phase on the polymorphism of PVDF, especially the relationship between miscibility and polymorphism, remains an open question and is not yet fully understood. In this work, three series of particle blends with varied levels of miscibility between PVDF and PMMA were prepared via seeded emulsion polymerization: PVDF–PMMA core–shell particle (PVDF@PMMA) with high miscibility; PVDF/PMMA latex blend with modest miscibility; and PVDF@c–PMMA (crosslinked PMMA) core–shell particle with negligible miscibility. The difference in miscibility, and the corresponding morphology and polymorphism were systematically studied to correlate the PMMA/PVDF miscibility with PVDF polymorphism. It is of interest to observe that the formation of polar β/γ phase during melt crystallization could be governed in two ways: dipole–dipole interaction and fast crystallization. For PVDF@PMMA and PVDF/PMMA systems, in which fast crystallization was unlikely triggered, higher content of β/γ phase, and intense suppression of crystallization temperature and capacity were observed in PVDF@PMMA, because high miscibility favored a higher intensity of overall dipole–dipole interaction and a longer interaction time. For PVDF@c–PMMA system, after a complete coverage of PVDF seeds by PMMA shells, nearly pure β/γ phase was obtained owing to the fast homogeneous nucleation. This is the first report that high miscibility between PVDF and PMMA could favor the formation of β/γ phase.

## 1. Introduction

The study on Poly(vinylidene fluoride)/Polymethylmethacrylate (PVDF/PMMA) binary blends has received significant attention in the past few decades. These semicrystalline-amorphous binary blends not only show miscibility on molecular level at all composition in the amorphous region, giving rise to a rich library of phase morphologies characteristics, but also combine advantageous physical properties from each individual polymer [1,2,3], namely, chemical resistance, flame-retardancy, toughness and electroactivity from PVDF, as well as excellent tensile strength, low smoke toxicity, and optical clarity from PMMA. Therefore, PVDF/PMMA blends are of technical importance in a broad number of potential applications. For instance, Zhang et al. reported that the presence of PMMA was effective to minimize charge accumulation and improve charge distribution in PVDF, delaying the apparatus failure when their blend was employed as low frequency cable [1]. Moreover, through facile control of PMMA content and membrane thermal history, PVDF/PMMA blends with optimized blood compatibility, hydrophilicity and processibility showed great promise for biomedical applications [2]. In addition, Li et al. studied the ferroelectric phase diagram of PVDF/PMMA and found the blends outperformed poly(vinylidene fluoride-*co*-trifluoroethylene) [P(VDF–TrFE)] copolymer above 100 °C, suggesting an ideal candidacy for the blends in high temperature application [3]. In fact, the physical properties of PVDF/PMMA blends have been well studied and closely related to their miscibility, the morphological structure of the blends, and the crystallization behavior of PVDF. Based on previous research, the miscibility pertained to the exothermic intermolecular interactions [4], and, in turn, had to do with the crystallization behavior of the blends [5]. Besides, the morphological structure was prominently determined by both miscibility and crystallization [6,7]. Therefore, from both scientific and technical points of view, it is extremely important to obtain a full understanding of the crystallization behavior of PVDF in a binary blend.

PVDF is a typical semicrystalline polymer exhibiting five crystal polymorphs, namely, α, β, γ, δ, and ε phases [8,9]. As a result, an enrichment of different electroactive properties can be identified in PVDF. Among them, the nonpolar α phase is the kinetically most favored phase. In each unit cell, two polymer chains in a trans-gauche (TGTG) conformation are packed anti-parallelly [10]. The δ phase has exactly the same chain conformation as that of the α phase, except two chains are arranged in parallel. Differently, the polar β phase exhibits the highest ferroelectric/piezoelectric responses owing to its all-trans zigzag conformation (TTTT) and two chains being arranged parallelly in each unit cell [11]. Polymer chains in both γ and ε phases twist at every fourth repeat units with a gauche conformation (T_3_GT_3_G′). The only difference between them is either parallel (γ) or anti-parallel (ε) chain arrangement [12]. It is reported that blending with PMMA could not only alter the kinetics of crystallization and the crystal morphology, but also lead to the variation in polymorphism (i.e., different crystal forms of PVDF). For the crystallization kinetics, the addition of PMMA would mainly influence the surface nucleation, because crystallization is controlled by two competitive rate-controlled processes: the attachment of crystalline polymer onto the crystal surface and the exclusion of amorphous polymer from the surface [13]. Different diffusion rate of the excluded PMMA chains may also lead to various spherulitic morphologies [7]. However, consistent and well-accepted conclusions regarding the effect of PMMA phase on PVDF crystal polymorphism have not yet been reached in academia. Direct crystallization from polar solvents or annealing right after melt quenching favored formation of a high percentage of β in PVDF/PMMA blends, whereas only marginal β phase was obtained even at high PMMA composition if the blend was prepared by melt mixing. It is clear from previous studies that in solvent crystallization, the solvent-PVDF chain interaction much more outweighed the PMMA–PVDF interaction in determining the final crystalline modification [14]. For melt quenching method, most of the primary samples were prepared by solution casting or spinning coating. Though the formation of β phase was ascribed to the increased trans-sequences and the reduced crystallization rate upon addition of PMMA, we consider that they may not be the main reason. This is because if the β phase was induced by PMMA, the relative content of β phase should increase as the PMMA content increases. In reality, the β phase content only increases at lower PMMA concentration (<20 wt %), and then decreases as more PMMA was added [3,14]. Since a portion of β phase could be obtained even in neat PVDF during quenching, it is possible that the change of polymorphism originated from the fast crystallization, similar to the ultrafast cooling in Gradys’ work [15] and confined crystallization in our previous work [16,17]. In a scenario that crystallization was directly elicited from polymer melts, sometimes, seemingly contradictory conclusions were drawn, no matter that polymer blends were prepared by either extrusion or solution mixing before hot pressing. For example, nearly pure α phase was obtained by Horibel et al. and Kim et al., even while PMMA contents were as high as 40 wt % and 50 wt %, respectively [18,19], whereas Fieire et al. could achieve 64% β phase at 40 wt % PMMA content, although neat PVDF showed 50% β phase using their preparation approach [20]. In addition, Zhang et al. observed the formation of β phase induced by PMMA in a PVDF/PMMA 70/30 blend [21]. Given the fact that the role of PMMA on PVDF polymorphism in a binary blend has not been well understood, it would be scientifically meaningful to investigate this interesting and molecularly miscible system, and clearly demonstrate the contribution of PMMA to PVDF polymorphism. Considering that most crystallization parameters, such as kinetics, morphology and melting temperature, are closely related to the PMMA/PVDF miscibility, we try to use the term of miscibility to explain PVDF polymorphism and crystallization behavior in PVDF/PMMA binary systems. To the best of our knowledge, relevant studies and interpretations have barely seen in the past literature, if not at all. 

In this work, we focused on the intercorrelation between the PMMA/PVDF miscibility and the change of PVDF polymorphism. Inspired by our previous work on PVDF@Polystyrene core–shell composite particles prepared via seeded emulsion polymerization [17,22], herein, we purposely designed three series of particle blends with varied levels of compatibility. From high to low miscibility, they are PVDF@PMMA core–shell composite particle, PVDF/PMMA latex blend and PVDF@c–PMMA (crosslinked PMMA) core–shell composite particle. As the main characterization methods, the miscibility difference was examined with differential scanning calorimetry (DSC), the crystal morphology was directly observed under polarized optical microscopy (POM) and the corresponding polymorphism was investigated by wide-angle X-ray diffraction (WAXD) and Fourier transform infrared (FTIR) spectroscopy. Compared to simple PVDF/PMMA latex blend, PMMA and PVDF chains in PVDF@PMMA were already intimately interacted at the interface of each core–shell particles, enabling a much larger biphasic area and a quicker inter-diffusion process for PMMA to interact with PVDF during heating and recrystallization. Therefore, higher amount of β phase was induced in the PVDF@PMMA particles. For the PVDF@c–PMMA particles, the chain inter-diffusion and mixing were largely impeded by the crosslinked, immobilized PMMA phase. The crystallization of PVDF was rarely influenced by PMMA phase until the PVDF seed particles were fully covered. When full coverage was reached, PVDF was able to undergo crystallization within a three-dimensional confined nano-space of crosslinked PMMA shell. As a result, a large portion of β phase was obtained due to fast homogeneous nucleation.

## 2. Experimental

### 2.1. Materials

Poly(vinylidene fluoride) (PVDF) latex, Kynar^®^ Latex 32, was kindly provided by Arkema Inc. (King of Prussia, PA, USA) Methyl methacrylate (MMA) and 1,4-butanediol diacrylate (BDDA) in analytically pure grade were purchased from Tianjin Reagent Corporation (Tianjin, China). Prior to use, the monomer MMA was distilled under reduced pressure (30 mmHg) at elevated temperatures (60 °C) to remove inhibitors. The crosslinking agent BDDA was used without any further purification. Potassium persulfate (K_2_S_2_O_8_) and sodium dodecyl sulfate (SDS) were purchased from Tianjin Kermel Chemical Reagent (Tianjin, China), and used without further purification. 

### 2.2. Sample Preparation

#### 2.2.1. Preparation of Core–Shell PVDF@PMMA Nanoparticles

The core–shell PVDF@PMMA colloidal particles were synthesized via seeded emulsion polymerization using PVDF latex particles as seeds. All polymerization reactions were carried out in a 250 mL four-necked round-bottom flask equipped with a condenser, a mechanical stirrer, a thermometer and an inlet for nitrogen. A determined amount of PVDF latex was introduced into the flask, and deionized (DI) water was added to make the total volume about 100 mL. The mixture dispersion was further ultrasonicated (using a 330 W ultrasonic bath, 50 Hz.) for 40 min to avoid any colloidal particle agglomeration. Then, nitrogen was purged into the dispersion for 15 min to remove O_2_ before polymerization, and fluxed during the entire polymerization. The monomer was added in one batch to swell the PVDF latex particles at room temperature for 40 min.

In this work, the feed ratio of MMA and PVDF was set at 4 to 1 in weight for making PMMA@PVDF particles. For PVDF@c–PMMA particles, a 4 wt % of BDDA relative to the amount of MMA monomer was added simultaneously with MMA to crosslink the grown PMMA shell during emulsion polymerization. The reaction mixture was then heated to 75 °C (or 65 °C) at a heating rate of 2 °C/min under a constant 300 rpm stirring. After an additional 15 min equilibration time, a 0.6 wt % of K_2_S_2_O_8_ relative to the amount of MMA monomer in 5 mL aqueous solution was added into the mixture. The polymerization time was varied from 0.5 to 6 h in order to obtain core–shell particles with different PMMA compositions. 

The obtained particles were purified by distillation below 40 °C under reduced pressure to remove unreacted monomer. An aliquot of sample was taken for morphology imaging and calculation of MMA conversion. Because the original PVDF weight was known before polymerization, the gained weight must come from the grown PMMA shell given that the MMA content in the condenser is negligible relative to the liquid phase.

#### 2.2.2. Preparation of PMMA Nanoparticles and PVDF/PMMA Blends

Neat PMMA latex particles were prepared using a similar way as the one discussed above. A 0.25 wt % of SDS relative to the amount of water was added into 130 mL of DI water in a 250 mL four-necked round-bottom flask. The solution was stirred at room temperature and nitrogen purging was maintained throughout the entire polymerization. After 15 min, 1.58 g of MMA monomer was transferred into the flask, followed by heating the mixture to 75 °C. Afterward, 10 mL 0.6 wt % K_2_S_2_O_8_ aqueous solution was added to initiate polymerization. An emulsion with translucent bluish color was seen in 1 h. Then, another aqueous solution, containing 0.6 wt % K_2_S_2_O_8_ of the MMA monomer, was added while 6.34 g of MMA was added dropwise at an adding rate of 2 g/min. After finishing feeding, the polymerization reaction was kept for another 2 h. The morphology and size distribution of PMMA latex particles are given in Supplementary Materials Figure S1.

Next, the PVDF latex and the prepared PMMA latex were blended together at room temperature, and the weight ratios of PVDF to PMMA latex particles were set at 70/30, 60/40 and 50/50 *wt*/*wt*, respectively. To achieve uniform dispersion, the mixture was kept stirred, followed by ultrasonication for 1 h. 

After mixing the above latexes, the resultant micro-particles were washed with DI water (3 × 20 mL), and collected by centrifugation. The collected micro-particles were dried overnight under reduced pressure at 40 °C. Table 1 collects the details of three series of samples, respectively.

### 2.3. Characterization

The particle morphology was imaged using a Hitachi Japan S-4800 scanning electron microscope (SEM, Tokyo, Japan). To make sample conductive, a thin layer of gold (10–30 Å) was sputter-coated under vacuum. The transmission electron microscope (TEM) images were collected by a JEOL Japan JEM-2010 FEF (Tokyo, Japan) transmission electron microscope. A drop of diluted latex solution was cast on 400-mesh carbon-coated copper grids and dried in the fume hood. The average size and size distribution of the core–shell particles were determined using a Malvern Zeta Sizer Nano 90 dynamic light scattering (DLS) particle size analyzer (Malvern, UK).

Thermal analysis was carried out using a Diamond differential scanning calorimeter (DSC, Perkin Elmer, Waltham, MA, USA). A modulated DSC mode was used to measure the crystallization temperatures (*T*_c_s) with about 7 mg load. Dry nitrogen was used as carrier gas. The DSC samples were heated from room temperature to 200 °C at a heating rate of 10 °C/min. The DSC cooling thermograms were obtained by annealing the samples for 10 min at 200 °C and cooling at a rate of −10 °C/min from 200 to 0 °C.

A polarized optical micrograph (POM) was obtained under crossed polarizers using a Zeiss Axioskop 40 (Zeiss, Jena, Germany). The spherulite morphology of the above-mentioned samples were examined with a hot stage. The samples were melted at 200 °C for 10 min, then isothermally crystallized at about 145 °C for 20 min.

Wide-angle X-ray diffraction (WAXD) was collected with a Bruker D8 Focus XRD diffractometer, using Cu Kα radiation (λ = 1.540 Å, the tube operated at 40 kV, the Bragg angle (2θ) in the range of 5–50°, with a scanning step rate of 4°/min).

Fourier transform infrared spectroscopy (FTIR) was conducted on a Bruker Vector 22 FTIR Spectrometer (Karlsruhe, Germany) in the spectral range of 4000–400 cm^−1^. The melt-recrystallized samples after DSC test were ground, and pressed into pellets with KBr powder.

## 3. Results and Discussion

### 3.1. Morphology and Size Distribution of Core–Shell Latex Particles

**Figure 1:**
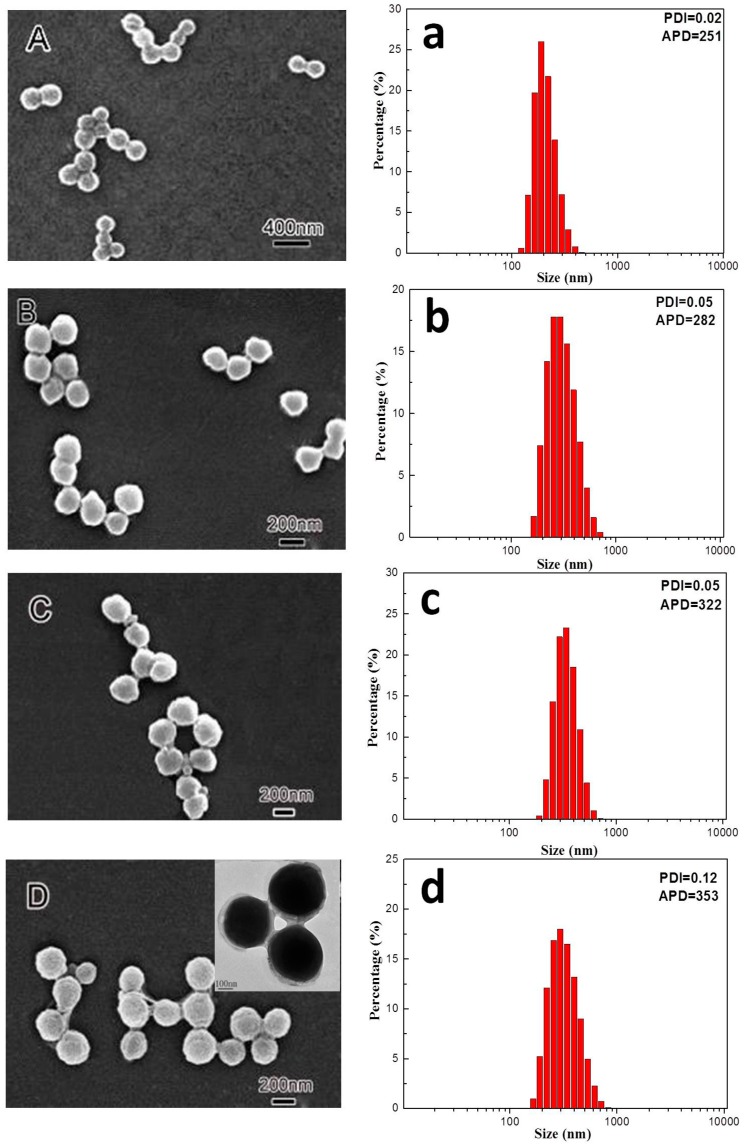
Scanning electron microscope (SEM) micrographs (**A**–**D**); and size distributions (**a**–**d**) of PVDF (Poly(vinylidene fluoride))@PMMA (Polymethylmethacrylate) series at the polymerization time of: (**A,a**) 0 h; (**B**,**b**) 0.5 h; (**C**,**c**) 1 h; and (**D**,**d**) 2 h. The inset in (**D**) is the transmission electron microscope (TEM) micrograph of the 2 h sample. APD means average particle diameter. PDI means polydispersity index.

The morphology evolution of the PVDF@PMMA core–shell nanoparticles during polymerization was studied by sampling aliquots at different time intervals. As shown in Figure 1, PVDF nanoparticles remained in regular spherical shape with relatively smooth surface after swollen by MMA monomer for 40 min (Figure 1A). The average particle diameter (APD) was around 251 nm with a narrow size distribution in which polydispersity index (PDI) was as low as 0.02 (Figure 1a). Starting from 30 min, larger particle size and slight irregular shape were seen from both SEM and DLS, indicating that MMA monomer started to polymerize and grow on PVDF latex surface. Considering that MMA is miscible with PVDF, some monomers likely diffused into the amorphous phase as well as the interface between PVDF and surfactant layer in the early swelling stage before seed polymerization. After polymerization, a penetrating PMMA/PVDF network might gradually form inside the PVDF latex. Meanwhile, the growing PMMA chains started to nucleate on the amorphous region on PVDF seed surface. Given that the crystallinity of PVDF was quite high (χ_c_ ~ 50%), PMMA might form numerous nucleation/growth sites all over the seed surface. This was essentially different from the growth of polystyrene, which can only form a bulge on PVDF surface because of poor compatibility [17,22]. With further increase of polymerization time to 1 h, the contour of latex particles became blurred. The average particles size and PMMA composition progressively increased. It suggested that particles were mostly covered with PMMA after 1 h of polymerization, and the PMMA layer was further thickened as reaction time increased. After reaction time reached 2 h, all PVDF latex particles were fully coated with uniform PMMA layers, as confirmed by both SEM and inset TEM images (Figure 1D). Generally, although the core–shell morphology of PVDF@PMMA nanoparticle could be confirmed by an electron microscope, the inner microstructure was very difficult to be exactly determined, however, considering the overall reaction process, the structure was mainly influenced by two factors: (1) the distribution of swollen MMA monomers along the radial direction inside the PVDF core; and (2) the inter-diffusion of PVDF and the polymerized PMMA. According to previous research, the inter-diffusion was mainly controlled by the diffusion of PMMA into PVDF when the temperature was below the melting point of PVDF [23,24]. Therefore, under the reaction temperature (75 °C, below the glass transition temperature of PMMA), the diffusion of PMMA into PVDF should be extremely small and could be negligible. Namely, the microstructure of the core–shell particle was mainly determined by the swollen MMA monomer. Since the PVDF latex was not dissolved by MMA, there should be a pure PVDF core inside the swollen layer. Based on the above discussion, we proposed that the core–shell particle composed of a pure PVDF inner layer, a gradual penetrated PMMA layer and a pure PMMA outer layer. The crosslinked core–shell particle (PVDF@c–PMMA) exhibited similar multi-layered structure as that of PVDF@PMMA particle, except for a chemically crosslinked PMMA outer layer.

In addition, it is noted that neither uncoated PVDF seeds nor isolated PMMA particle was identified in the final core–shell product, as convinced from the DLS results that the particles with a relatively narrow PDI value were finally obtained. This indicated that nano-sized PVDF latex efficiently acted as primary seeds to enable surface growth of PMMA via emulsion polymerization, presumably due to its large-area surface availability as well as great compatibility to MMA monomer and polymer. It was also corroborated by theoretical and experimental results reported in the past literature [25,26,27,28].

### 3.2. PVDF/PMMA Miscibility in Different Complexation Scenarios

**Figure 2:**
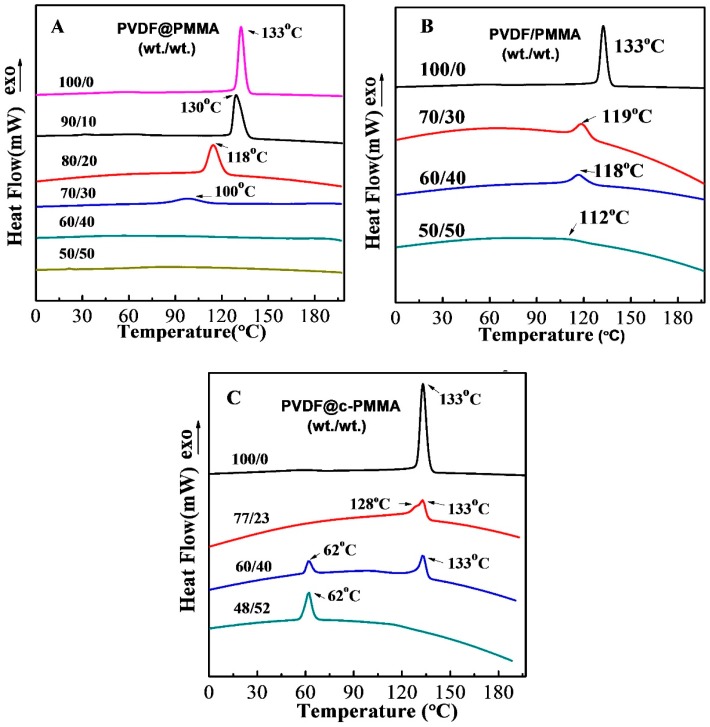
DSC cooling curves showing the crystallization traces of three series of the samples: (**A**) PVDF@PMMA; (**B**) PVDF/PMMA; and (**C**) PVDF@c–PMMA, at various weight ratios of PVDF to PMMA. The cooling rate was −10 °C/min.

The non-isothermal crystallization behaviors of PVDF@PMMA, PVDF@c–PMMA and PVDF/PMMA samples with varied PMMA contents were studied by DSC analysis, as shown in Figure 2. It is clear that *T*_c_ of PVDF gradually decreased as increasing PMMA content in both PVDF@PMMA and PVDF/PMMA samples, indicating a significant slow-down of crystal growth rate. It was usually attributed to the suppressed PVDF segments mobility caused by the intermolecular interactions between the carbonyl group of PMMA and the hydrogen of PVDF [5,29]. Compared to PVDF/PMMA, more significant suppression effect of PMMA was found in PVDF@PMMA. The *T*_c_ of PVDF in PVDF@PMMA decreased from 133 to 100 °C as the PMMA composition increased from 0 to 30 wt %. Nonetheless, it only decreased to 119 °C in PVDF/PMMA. Further increasing the PMMA content (up to 40 wt %), the exothermic peak disappeared in PVDF@PMMA while a relatively strong peak can still be identified in the normal blend (*T*_c_ = 118 °C, only 15 °C lower than pure PVDF). The crystallization peak in PVDF/PMMA remained discernable until the PMMA content was higher than 50 wt %. Similar effect was also observed during the first heating process, as shown in Supplementary Materials, Figure S2A,B. The melting temperature (*T*_m_) of PVDF gradually decreased from 160 to 149 °C when the weight ratio of PVDF@PMMA decreased to 60/40, while only little change in *T*_m_ (159 °C) were traced in PVDF/PMMA. The above DSC evidence suggested a stronger intermolecular interaction between PVDF and PMMA in PVDF@PMMA during crystallization. Note that the particular core–shell morphology in PVDF@PMMA featured an enormously large PMMA–PVDF interfacial area and pre-interwoven polymer chains at interface. This presumably led to an enhanced inter-diffusion thereafter starting from the interfacial area during the first heating. In other words, when temperature increased above both *T*_g_ of PVDF and PMMA, the inter-diffusion was so efficient in PVDF@PMMA that a much greater extent of PMMA–PVDF chain intermixing at finer length scale could be resulted. Therefore, PVDF and PMMA polymer chains were more intimately interwoven together, leaving more carbonyl groups on PMMA and more hydrogens on PVDF readily interacting with each other during cooling and recrystallization. In contrast, physically isolated PMMA and PVDF phases stayed in their latex particles in normal blend sample with very limited contact area, which means that it must be more difficult for normal latex blend to reach the same length scale of chain intermixing as that in the core–shell sample at a given heating time. Coarse scale of chain intermixing would somewhat make carbonyl groups and hydrogens less accessible to each other. As a result, the crystallization of some PVDF chains might be unaffected by PMMA, and hence a weaker crystallization suppression was observed in the normal blends.

In contrast, the PVDF@c–PMMA exhibited totally distinct crystallization behavior. From the first cooling curve (see Figure 2), there was a weak shoulder peak centered at 128 °C in company with the main peak at 133 °C in PVDF@c–PMMA 77/23, this was quite different from the largely suppressed crystallization for PVDF@PMMA 80/20. Because of the formation of crosslinked network in PMMA, the chain mobility and inter-diffusion were restricted, only the PVDF chains close to c–PMMA domains could be influenced by PMMA, most of PVDF chains were free to crystallize as in the bulk. Intriguingly, with the increased concentration of c–PMMA (40 wt %), fractionated crystallization was observed with a major crystallization peak at 133 °C and a minor crystallization peak at 62 °C. Note that the similar crystallization temperature *T*_c_ was observed within 60–65 °C in well confined nanodroplets and PVDF@PS composite latex from our previous work [16,17]; therefore, a portion of PVDF particles were fully wrapped by c–PMMA shells, which provide confined nano-space for homogenously nucleated crystallization. Finally, the crystallization peak at 133 °C almost disappeared (52 wt % c–PMMA, Figure 2C), and a distinct homogeneous crystallization peak located at 62 °C, demonstrating that all PVDF particles were well confined by c–PMMA shells.

**Figure 3:**
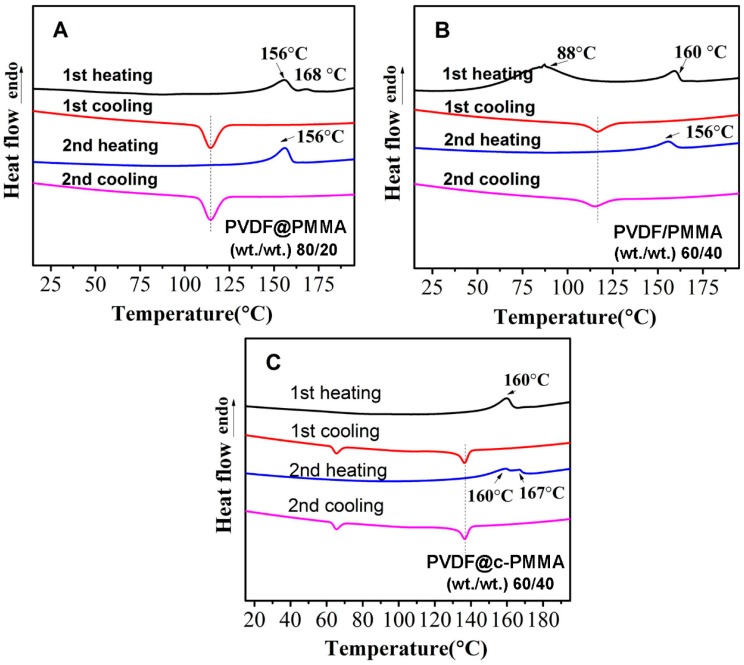
Heating–cooling cycle curves for selected PMMA contents in different latex blends: (**A**) PVDF@PMMA 80/20; (**B**) PVDF/PMMA 60/40; and (**C**) PVDF@c–PMMA 60/40.

To further understand the difference, the heating–cooling cycle data for selected composition are shown in Figure 3. Since the maximum crystallization peak for PVDF@PMMA 80/20 and PVDF/PMMA 60/40 were found at nearly the same position, they were selected for comparison. In PVDF@PMMA, it is clear that the first and second heating/cooling curves were nearly identical except that a small γ peak (*T*_m_ = 168 °C) only appeared on the first heating curve. This indicated that in PVDF@PMMA, the inter-diffusion and chain mixing quickly proceeded in the amorphous regions at relatively low temperature (<*T*_m_) during the first heating. The PVDF crystals were surrounded by perfectly mixed PMMA–PVDF composite phase, and the melting temperature was suppressed during the first heating. Contrarily, in PVDF/PMMA 60/40, besides the main melting peak equal to neat PVDF (centered at 160 °C) another distinct endothermic peak centered close to 90 °C was observed in first heating curve, which has previously been ascribed to melting of some small, imperfect PVDF crystallites [3,30]. During second heating, the main melting peaks decreased to 156 °C while the other endothermic peak disappeared (Figure 3A,B). As for the first and second cooling process, we could find that they show same exothermic behavior (at 118 °C), which meant that inter-diffusion and chain intermixing in normal blend was not fast and proceeded along with the PVDF crystal melting, exerting minimal influence on the *T*_m_ during the first heating. After the first heating (*T* > *T*_m_), PVDF and PMMA in normal blend had better mixing and interfacial area than they were before. During the second heating, the *T*_m_ of PVDF started to be affected by PMMA. Therefore, PVDF@PMMA could reach to equal chain intermixing more efficiently than PVDF/PMMA, Meanwhile, the same melting and crystallization temperature (i.e., second heating and second cooling) for PVDF@PMMA 80/20 and PVDF/PMMA 60/40 revealed that less PMMA in PVDF@PMMA could provide same influence on the crystallization kinetics, referring to the better mixing of PMMA due to larger interfacial area.

Keeping in line with the re-crystallization behavior, the melting behavior of PVDF@c–PMMA 60/40 was also distinctive. Compared with the previous two miscible series, three major differences should be addressed. First, the melting point of PVDF was not much influenced in the first heating scan (Figure 3C), indicating that PMMA was successfully crosslinked during the synthesis process, preventing the diffusion of PMMA into PVDF. Second, the re-crystallization peaks during the first and second cooling curves appeared at the exact same position, further confirming the obstruction of interchain diffusion between PVDF and PMMA. Third, double melting peaks were tracked in second heating process, i.e., the lower peak centered at 160 °C was ascribed to α phase from the bulk crystallization, and the higher peak centered at 167 °C was related to the β phase from the confined crystallization, in consistence with the fractionated crystallization behavior, which will be discussed in the following part. 

### 3.3. The Morphology of PVDF Crystal in Different Complexation Scenarios

**Figure 4:**
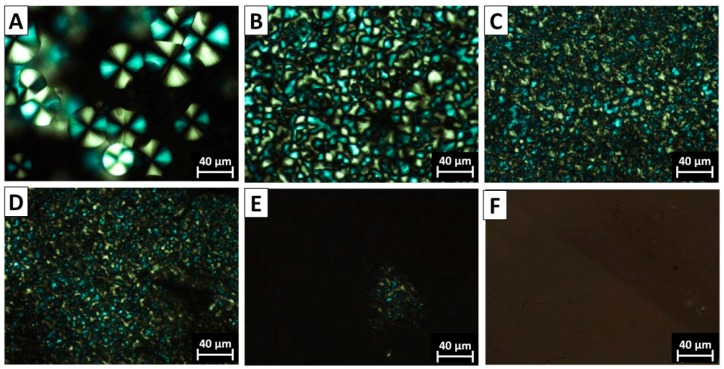
Polarized optical micrograph (POM) images for the PVDF@PMMA samples with the core–shell weight ratio of: (**A**) neat PVDF; (**B**) 90/10; (**C**) 80/20; (**D**) 70/30; (**E**) 60/40; and (**F**) 50/50.

**Figure 5:**
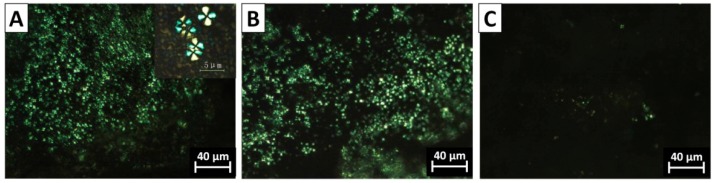
POM images of the PVDF/PMMA samples with the weight ratio of: (**A**) 70/30; (**B**) 60/40; and (**C**) 50/50.

**Figure 6:**
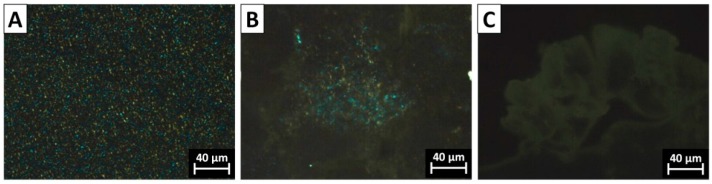
POM images of the PVDF@c–PMMA samples with the weight ratio of: (**A**) 77/23; (**B**) 60/40; and (**C**) 48/52.

The spherulite crystal morphology clearly speaks of the diversity of chain intermixing in different complexation/blending scenarios. POM images of typical crystal morphologies of neat PVDF and the PVDF@PMMA samples with different weight ratios are shown in Figure 4. In neat PVDF, compact spherulites with a clear Maltese-cross pattern were observed (Figure 4A). The average diameter of PVDF spherulites was above 30 μm. In PVDF@PMMA, the presence of PMMA drastically increases the density of nuclei, thus the viewing field was full of numerous smaller spherulites, as seen in Figure 4B–D. Meanwhile, the Maltese-cross pattern gradually vanished, along with the appearance of coarse spherulites whose lamellar bundles showed large cross section. When the weight ratio reached 50/50, no spherulite structure was visible in PVDF@PMMA, further confirming an excellent chain mixing between PVDF and PMMA, i.e., PMMA and PVDF chains were homogenously mixed with each other, which prevented formation of PVDF-rich phases, and hence ordered crystal structures. Similarly, the crystal morphology evolution in PVDF/PMMA blend exhibited the same trend as that of PVDF@PMMA. In Figure 5, PVDF spherulites with dim Maltese-cross pattern were less uniformly distributed in PVDF/PMMA 70/30 and 60/40 samples. However, a few spherulites could still be identified in PVDF/PMMA 50/50 sample, but not in PVDF@PMMA 50/50 sample. The difference should be ascribed to a less efficient chain mixing in the normal blend, which is consistent with the higher *T*_c_, as seen in previous DSC results (Figure 2B). In contrast, owing to the restricted mobility of PMMA in PVDF@c–PMMA nanoparticle (Figure 6), the morphology evolution is completely different from the other two samples. If most particles were not well covered by c–PMMA, isolated c–PMMA domains may act as nucleation sites, thus numerous uniform and smaller lamellar bundles were observed (Figure 6A). As the c–PMMA content increased, a portion of PVDF particles was fully covered. The smaller spherulites were not uniformly distributed in the viewing field, and some gel-like structure appeared. When 50/50 ratio was reached, there were no Maltese-cross patterns visible under microscope, but rather a gel-like network that indicated the PVDF phase was completely confined by the c–PMMA shell.

### 3.4. Polymorphic PVDF Crystallization Induced in Different Complexation Scenarios

**Figure 7:**
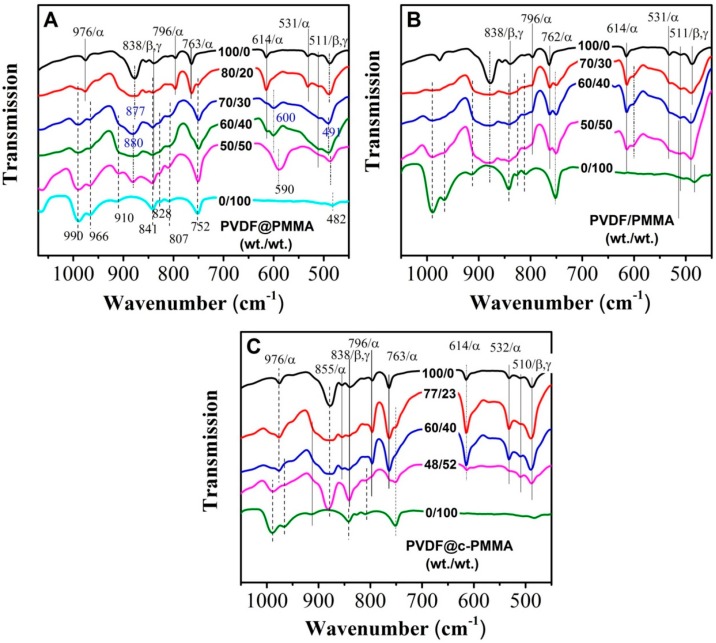
Fourier transform infrared spectroscopy (FTIR) profiles of three series of the samples: (**A**) PVDF@PMMA; (**B**) PVDF/PMMA; and (**C**) PVDF@c–PMMA, at various weight ratios.

**Figure 8:**
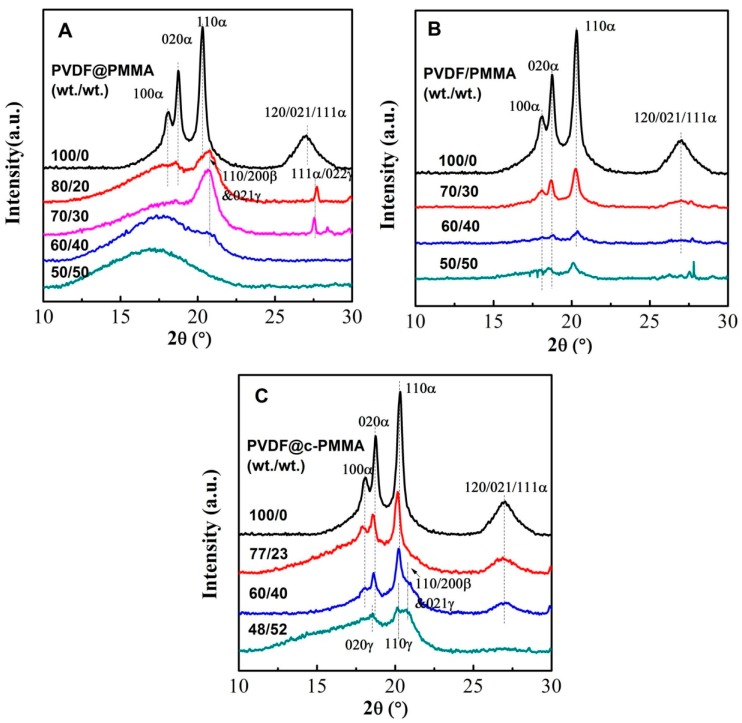
Wide-angle X-ray diffraction (WAXD) profiles of three series of the samples: (**A**) PVDF@PMMA; (**B**) PVDF/PMMA; and (**C**) PVDF@c–PMMA, at various weight ratios.

It is well known that the van der Waals volume of fluorine and hydrogen atom is similar, which results in a more flexible PVDF chain conformation and a rich crystal polymorphism. A comparative study of crystal isomorphism for the three blending scenarios was carried out by FTIR and WAXD, aiming to understand how chain intermixing between PVDF and PMMA would affect the crystal structure of PVDF. FTIR and WAXD results are plotted in Figure 7 and Figure 8, respectively. For neat PVDF, the intensive absorption peaks for TGTG’ conformation (i.e., 531/α, 614/α, 763/α, 796/α, and 976/α cm^−1^) along with several relatively weak peaks for TTT conformation (511/β,γ, and 838/β,γ cm^−1^) were observed, indicating the formation of large amount of α phase [11,31,32]. It was also confirmed by the diffraction peaks of α phase in the XRD as well as the deconvoluted peaks in Supplementary Materials, Figure S3A, i.e., 100α, 020α and 110α [8,33,34]. For PVDF@PMMA 80/20, although the FTIR pattern was somewhat similar to that of neat PVDF (i.e., the appearance of 531/α, 614/α, 763/α, 796/α, 976/α, 511/β,γ, and 838/β,γ cm^−1^) with two more peaks from amorphous PMMA (i.e., 752 and 990 cm^−1^), their WAXD profiles looked very differently. The intensity of 100α and 020α peaks decreased dramatically, and 110α peak shifted to high angle. From the deconvolution curves, it is clear that both the diffraction peaks of α (100α, 020α, and 110α) and β phase (110/200 β) were detected, suggesting the coexistence of α and β crystals. Comparing the WAXD pattern of neat PVDF and PVDF@PMMA 80/20, we assume that only α phase was formed in neat PVDF. The weak signal of the TTT conformation in FTIR may result from external force-induced local conformation change during the grinding process for making the testing pellet. This is not unusual, since similar a phenomenon was also observed when examining the cutting surface of PVDF resin using attenuated total reflection (ATR) mode. Thus, the observed TTT conformation was mainly artifact. With increasing PMMA content in PVDF@PMMA, characteristic IR peaks attributed to PMMA [29,35] (i.e., 752, 807, 828, 910, 966, 990 cm^−1^) became stronger, as marked by dot line in Figure 7A. In contrast, the reflection peaks belonging to PVDF crystal either totally disappeared (i.e., 531/α, 763/α, 796/α cm^−1^) or became extremely weak (511/β, γ, 614/α cm^−1^) and indistinguishable (838/β, γ cm^−1^). It is worth to mention that an IR absorption peak centered at 600 cm^−1^ became noticeable when the PMMA content was higher than 30 wt %, and it shifted to 590 cm^−1^ in the PVDF@PMMA 50/50 sample after re-crystallization. This new peak can neither be indexed to any peak associated with amorphous PMMA, nor to any forms of PVDF crystal structures. At 50/50 weight ratio, only amorphous blend was obtained as confirmed by WAXD. Thus, we speculate that it may be related to the amorphous PVDF, given the fact that this peak is also visible in PVDF melts [36]. In addition, its peak intensity would increase when α to β phase transition happened during PVDF stretching, since that crystallinity greatly reduced during stretching, leaving more amorphous PVDF phase [37,38]. Considering that the signal of FTIR was significantly interfered by PMMA, WAXD was used to determine the polymorphic structures. As shown in Figure 8A, the intensity of the diffraction peaks (i.e., 100α, 020α, 110α, 120/021/111α) of PVDF α phase decreased significantly due to the addition of PMMA. Instead, a broad peak appeared at 20.75°, which may be ascribed to the overlap of the diffraction peaks of 110α and 110/200β or 021γ. To distinguish each diffraction peak associated with different crystal phases, Gaussian fitting was employed to deconvolute the overlapped area, as shown in Supplementary Materials, Figure S3A–L. For PVDF@PMMA sample, the percentage of polar β/γ phases among the total crystal phases increased as the PMMA content increased from 20 wt % to 30 wt %, i.e., the intensity ratio of 110/200β and 021γ to 110α at 20.2° increased from 1.39 to 1.69 (see Table 2). Nonetheless, the crystallinity dropped from 23.85% to 18.06%. Note that the amorphous peak area contained the contribution from misicible PMMA, therefore, the calculated crystallinity may be lower compared with the actual value. When PMMA content reached 40 wt %, only a weak diffraction peak (110/200β and 021γ) was observed on the shoulder of the diffused amorphous peak, and the crystallinity further decreased to 3.57%. Above 40 wt %, only a amorphous halo could be detected. Therefore, β phase was preferentially formed at the expense of total crystallinity during re-crystallization in PVDF@PMMA. For PVDF/PMMA normal blend, three types of IR peaks, including multiple peaks belonging to TGTG’ conformation of α phase (i.e., 531, 614, 762, and 796 cm^−1^), two peaks associated with TTT conformation of β or γ phase (i.e., 511 and 840 cm^−1^), and a weak amorphous PVDF peak (600 cm^−1^), were seen (Figure 7B). The FTIR pattern seems not very sensitive to the change of PMMA content in PVDF/PMMA sample, as they all looked similar. While WAXD results showed that the portion of polar β phase modestly increased as the PMMA content increased (i.e., the intensity ratio between selected β and α diffraction peaks went up from 0.17 to 0.34), α phase still accounted for the main part as revealed from the X-ray diffraction (Figure 8B). This implied that polar PVDF β phase could be more easily induced in PVDF@PMMA sample than it was in PVDF/PMMA sample. Moreover, the crystallinity in PVDF/PMMA was much higher than that in PVDF@PMMA (18% vs. 45% at 30 wt % PMMA content). Therefore, PVDF crystallization behavior was much less affected in PVDF/PMMA owing to insufficient PMMA/PVDF chain mixing. The inter-chain spacing of neat PVDF latex and PVDF/PMMA 70/30 was almost the same, while they were both shorter than that of PVDF@PMMA 70/30 (see in Table 2). It means that neighboring PVDF chains were placed further apart from each other with weaker interaction or van der Waals force, as miscible PMMA chain might sneak in between and weaken the interaction. 

Intriguingly, because the diffusion of PMMA was prohibited by the crosslinked network, the polymorphous behavior of PVDF@c–PMMA was totally different from the other two samples. From the FTIR spectrum of PVDF@c–PMMA 77/23, we observed some strong absorption peaks from TGTG’ conformation (i.e., 532/α, 614/α, 763/α, 796/α, 976/α cm^−1^) together with relatively weak peaks from TTT conformation (511/β,γ, 838/β,γ cm^−1^), indicating a combination of major α phase and very minor β/γ polar phase. The deconvoluted diffraction peaks corresponding to 110/200β and 021γ (2θ = 20.8°) are well documented in Supplementary Materials, Figure S3J, and corroborated FTIR results. The formation of a small amount of β phase should be ascribed to the dipole–dipole interaction at the interface between isolated c–PMMA domain and PVDF, because the interdiffusion was unlikely to happen, which was also confirmed by the almost invariable average intrachain separation and crystallinity in PVDF@c–PMMA (Table 2). At higher PMMA content (e.g., 60/40), more β/γ phase was obtained. As a result, the 510 cm^−1^ β/γ peak in FTIR became more intense, and the 110/200β and 021γ diffraction peak could be well identified from the original WAXD profile. The induced β/γ phase primarily originated from the confined crystallization of PVDF that was fully covered by c–PMMA shell. An extra homogeneous crystallization peak at 62 °C, together with a normal crystallization peak at 133 °C, was clearly seen in DSC. With further increasing the PMMA content, almost all peaks assigned to TGTG’ conformation disappeared in FTIR. Meanwhile, only the diffraction peaks of β/γ phase could be seen in the original WAXD profile, indicating that nearly pure polar PVDF phase was achieved in each core–shell particle. Here, although there may be some contribution from the polar confined environment, since polar substrate also could induce the β/γ phase, it was not the main reason because fast homogenous nucleation would limit the influence of dipole–dipole interaction.

It is worth stressing that those PVDF@c–PMMA particles might be promising as novel ferroelectric polymers in electrical energy storage. Compared to our previous work [17], i.e., PVDF@PS core–shell latex particle (~20 wt % PVDF), higher PVDF content with polar β phase was obtained in PVDF@c–PMMA (~50 wt % PVDF), owing to the good miscibility between MMA monomer/polymer and PVDF during emulsion polymerization. Unlike PS that is immiscible with PVDF, 1–3 discrete PS bulges first form on the surface of PVDF seeds at the early stage of polymerization, while MMA could randomly form a great number of nucleation sites in the beginning, isolated PMMA domains will soon meet each other and develop to continuous shell and cover PVDF seeds. Less reaction time means thinner PMMA shell and lower PMMA content for full surface coverage. It is well known that bulk PVDF is a good ferroelectric polymer with attractively high dielectric constant. However, micron size or even larger ferroelectric domains in bulk PVDF resulted in huge dielectric hysteresis and loss, which is not acceptable in any commercial dielectric capacitor. It is well proposed that breaking large ferroelectric domains into nano-domains could suppress dielectric hysteresis by minimizing strong dipole–dipole interaction, and shift traditional ferroelectrics into relaxor ferroelectrics. Thus, core–shell morphology is one of the ideal structures for achieving such nano-domain structure, since PMMA or PS shell could act as the barrier to prevent the formation of large ferroelectric domains. Therefore, PVDF inside each core–shell particle will at most comprise a couple of nano-domains, if not a single one. As a result, dielectric hysteresis and loss will be significantly reduced under remaining high dielectric constant. If PVDF content in core–shell particles is low, the whole materials will lose high dielectric constant, and ferroelectric behavior. Therefore, only core–shell structure with high PVDF content is desirable for high dielectric constant and low hysteresis. If, in the future, we could further increase the PVDF content by optimizing polymerization condition or decrease PVDF domain size by using smaller seed particles, PVDF@c–PMMA should be a more cost-efficient relaxor ferroelectric polymer composites compared to expensive PVDF terpolymers such as poly(vinylidene fluoride-*co*-trifluoroethylene-*co*-chlorotrifluoroethylene) (P(VDF–TrFE–CTFE)) [39].

### 3.5. Structure Evolution in Different Complexation Scenarios

**Figure 9:**
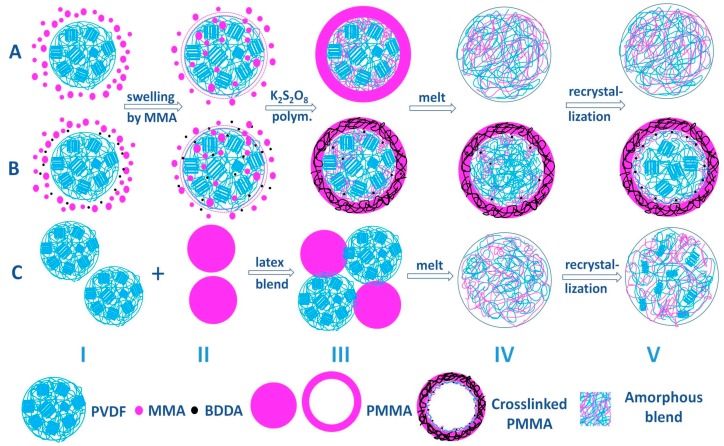
Schematic of the preparation of latex blends and their structure evolution during melt and re-crystallization: (**A**) PVDF@PMMA; (**B**) PVDF@c–PMMA; and (**C**) PVDF/PMMA.

Based on the previous results and discussions, a schematic is given in Figure 9 to illustrate how three complexation scenarios evolve in structure and morphology, from the beginning of preparation to the final re-crystallization. For the core–shell particles, in the monomer swelling stage before the seeded emulsion polymerization, the PVDF latex particle is stabilized by the existing surfactants on the PVDF seed. Because MMA is miscible with PVDF, two processes would happen with the increasing of swelling time: (1) a thin layer of MMA monomer (with or without BDDA) forms between the PVDF particle and the existing surfactant layer; and (2) MMA (and BDDA) wets the surface and further diffuses into the PVDF seed particle (Figure 9A,B, Stages I–II). As the initiator was added, the polymerization was initiated, the coated polymer layer was gradually produced with the polymerization proceeding. Inside the seed particles, the entrapped monomer formed penetration network with physical (Figure 9A, Stage III) or chemical (Figure 9B Stage III) entanglement in the amorphous region of PVDF. As a result, the PVDF@PMMA leads to a better dispersion in microscale during the melting process (Figure 9A, Stage IV), which assures the stronger interactions between the molecular chains, resulting in the full suppression of crystallization under high PMMA content (Figure 9A, Stage V). In PVDF@c–PMMA, the chemical crosslink blocked the diffusion of PMMA into PVDF (Figure 9B, Stage IV), leading to homogenous nucleation in a confined 3D nano-sphere (Figure 9B, Stage V).

For PVDF/PMMA, the diffusion of PMMA to PVDF was triggered after the latex going through ultrasonic mixing procedure, the pre-mixed part before melting was fairly limited (Figure 9C, Stage III), therefore the intermixing was not as better as in PVDF@PMMA. As a result, after melting re-crystallization, there were still some smaller lamellas observed in mixed amorphous matrix with 50 wt % PMMA (Figure 9C, Stage V, also see Figure 5).

## 4. Conclusions

By employing emulsion polymerization of MMA in the presence of PVDF latex seeds, three series of latex blends featuring different levels of miscibility between PVDF and PMMA were designed and prepared. Thereafter, the crystallization behavior of PVDF in these blends was systematically studied. In miscible systems (PVDF@PMMA and PVDF/PMMA), the *T*_c_ of PVDF shifted to lower temperature and the crystallization peak gradually vanished with a decreased peak intensity as the PMMA content increased. Meanwhile, higher content of β phase with lower crystallization capacity was obtained, indicating that the crystallization of PVDF was significantly influenced by PMMA because of the intermolecular interactions. Moreover, at the same PMMA content, PVDF in PVDF@PMMA blends exhibited higher percentage of β phase and lower crystallization capacity than in PVDF/PMMA. In other words, the crystallization behavior of PVDF was more affected by PMMA in PVDF@PMMA. We attributed this to a better PMMA/PVDF chain intermixing or miscibility in PVDF@PMMA, as also evidenced by a more pronounced suppression of crystallization and a larger interchain spacing between neighboring PVDF chains. To the best of our knowledge, this is the first report that high miscibility between PVDF and PMMA could favor the formation of polar β/γ phase. On the contrary, the crystallization behavior of PVDF in PVDF@c–PMMA is much different from the cases in the other two blends, due to a crosslinked and immobile PMMA phase. When PVDF seeds were not fully wrapped by PMMA, the *T*_c_ and the crystal polymorphism were slightly changed, which mainly originated from the dipole–dipole interactions between PVDF and isolated PMMA domains. After a full PMMA coverage was achieved, the nanoscale confinement led to an exclusive homogeneous crystallization, resulting in the formation of β/γ phase. Moreover, the PMMA shell would break large ferroelectric domains into nano-domains, resulting in novel relaxor ferroelectric behavior with high dielectric constant and low hysteresis, which is desirable for various applications such as energy storage, electrocaloric cooling, electrostrition, and so on.

## Figures and Tables

**Table 1:** The feeding ratio, reaction time, reaction temperature and composition of three complexation scenarios.

Sample series	PVDF/MMA ratio (*wt*/*wt*)	Polymerization temperature (°C)	Reaction time (h)	Composition ratio of PVDF to PMMA (*wt*/*wt*)
PVDF@PMMA	1:4	75	0.5	70/30
	1:4	75	1	60/40
	1:4	75	2	50/50
PVDF@c–PMMA	1:5	65	4	77/23
	1:5	65	5	60/40
	1:5	65	6	48/52
PVDF/PMMA ^1^	7:3			70/30
	6:4			60/40
	5:5			50/50

^1^ MMA monomers were polymerized at 75 °C. The solid contents of the PVDF and the PMMA latexes were 19.5 wt % and 7.2 wt %, respectively.

**Table 2:** Some parameters obtained from X-ray deconvolution curves.

Sample	Composition ratio (*wt/wt*)	Area ratio (110/200)β/110α	Amorphous peak position (2θ/°)	Average interchain separation (Å) ^1^	Crystallinity ^2^ (%)
PVDF latex	100/0	0	19.09	5.81	38.20
PVDF@PMMA	80/20	1.39	17.99	6.16	23.85
	70/30	1.69	17.93	6.19	18.06
	60/40	----	17.56	6.32	3.57
	50/50	----	16.90	6.56	0
PVDF/PMMA	70/30	0.17	18.99	5.84	45.00
	60/40	0.34	18.67	5.94	32.42
	50/50	0.33	18.07	6.14	23.86
PVDF@c–PMMA	77/23	0.16	16.4/19.09	5.81	27.52
	60/40 ^3^	1.16	19.09	----	28.34
	48/52	----	15.2/19.10	5.80	24.47

^1^ The average interchain separation was calculated using the equation <R> = 5/8 (λ/sinθ), details are shown in Ref. [40]. ^2^ In PVDF@PMMA and PVDF/PMMA series, the crystallinity was calculated using the whole amorphous area, including the contribution from PMMA, in PVDF@c–PMMA, amorphous peak centered at about 19.09° was used. ^3^ The sample could not be well fitted to get a meaningful interchain separation value.

## Supplementary Materials

The following are available online at www.mdpi.com/2073-4360/9/9/448/s1, Figure S1: (A) SEM image; and (B) size distribution of PMMA latex particles. Figure S2: DSC first heating curves of: (A) PVDF@PMMA; and (B) PVDF/PMMA at various weight ratios of PVDF to PMMA. The cooling rate was −10 °C/min. Figure S3: Deconvolution of the X-ray diffraction curves for: (A) neat PVDF; (I) neat PMMA; and three series of samples: (B–E) PVDF@PMMA; (F–H) PVDF/PMMA; and (J–L) PVDF@c–PMMA.

## References

[1] Lei C., Wang X., Tu D., Wang H., Du Q. (2009). Charge distribution in PVDF/PMMA blends under DC field. Mater. Chem. Phys..

[2] Ai F., Li H., Wang Q., Yuan W.Z., Chen X., Yang L., Zhao J., Zhang Y. (2012). Surface characteristics and blood compatibility of PVDF/PMMA membranes. J. Mater. Sci..

[3] Li M., Stingelin N., Michels J.J., Spijkman M.-J., Asadi K., Feldman K., Blom P.W.M., Leeuw D.M. (2012). Ferroelectric phase diagram of PVDF: PMMA. Macromolecules.

[4] Fan W., Zheng S. (2007). Miscibility and crystallization behavior in blends of poly (methyl methacrylate) and poly (vinylidene fluoride): Effect of star-like topology of poly (methyl methacrylate) chain. J. Polym. Sci. B.

[5] Sasaki H., Bala P.K., Yoshida H., Ito E. (1995). Miscibility of PVDF/PMMA blends examined by crystallization dynamics. Polymer.

[6] Luciani A., Jarrin J. (1996). Morphology development in immiscible polymer blends. Polym. Eng. Sci..

[7] Okabe Y., Murakami H., Osaka N., Saito H., Inoue T. (2010). Morphology development and exclusion of noncrystalline polymer during crystallization in PVDF/PMMA blends. Polymer.

[8] Tashiro K. (1995). Crystal structure and phase transition of PVDF and related copolymers. Plast. Eng. N. Y..

[9] Lovinger A.J. (1982). Annealing of poly (vinylidene fluoride) and formation of a fifth phase. Macromolecules.

[10] Takahashi Y., Matsubara Y., Tadokoro H. (1983). Crystal structure of form II of poly (vinylidene fluoride). Macromolecules.

[11] Kobayashi M., Tashiro K., Tadokoro H. (1975). Molecular vibrations of three crystal forms of poly (vinylidene fluoride). Macromolecules.

[12] Takahashi Y., Tadokoro H. (1980). Crystal structure of form III of poly (vinylidene fluoride). Macromolecules.

[13] Saito H., Okada T., Hamane T., Inoue T. (1991). Crystallization kinetics in mixtures of poly (vinylidene fluoride) and poly (methyl methacrylate): Two-step diffusion mechanism. Macromolecules.

[14] Kim K.J., Cho Y.J., Kim Y.H. (1995). Factors determining the formation of the β crystalline phase of poly (vinylidene fluoride) in poly (vinylidene fluoride)-poly (methyl methacrylate) blends. Vib. Spectrosc..

[15] Gradys A., Sajkiewicz P., Adamovsky S., Minakov A., Schick C. (2007). Crystallization of poly (vinylidene fluoride) during ultra-fast cooling. Thermochim. Acta.

[16] Zhong G., Wang K., Zhang L., Li Z.-M., Fong H., Zhu L. (2011). Nanodroplet formation and exclusive homogenously nucleated crystallization in confined electrospun immiscible polymer blend fibers of polystyrene and poly (ethylene oxide). Polymer.

[17] Pan M., Yang L., Wang J., Tang S., Zhong G., Su R., Sen M.K., Endoh M.K., Koga T., Zhu L. (2014). Composite poly (vinylidene fluoride)/polystyrene latex particles for confined crystallization in 180 nm nanospheres via emulsifier-free batch seeded emulsion polymerization. Macromolecules.

[18] Horibe H., Hosokawa Y., Oshiro H., Sasaki Y., Takahashi S., Kono A., Nishiyama T., Danno T. (2013). Effect of heat-treatment temperature after polymer melt and blending ratio on the crystalline structure of PVDF in a PVDF/PMMA blend. Polym. J..

[19] Lee M., Koo T., Lee S., Min B.H., Kim J.H. (2015). Morphology and physical properties of nanocomposites based on poly (methyl methacrylate)/poly (vinylidene fluoride) blends and multiwalled carbon nanotubes. Polym. Compos..

[20] Freire E., Bianchi O., Martins J.N., Monteiro E.E., Forte M.M.C. (2012). Non-isothermal crystallization of PVDF/PMMA blends processed in low and high shear mixers. J. Non-Cryst. Solids.

[21] Li W., Li H., Zhang Y.-M. (2009). Preparation and investigation of PVDF/PMMA/TiO2 composite film. J. Mater. Sci..

[22] Pan M., Yang L., Guan B., Lu M., Zhong G., Zhu L. (2011). Surface nucleation-induced fluoropolymer Janus nanoparticles via emulsifier-free batch-seeded emulsion polymerization. Soft Matter.

[23] Lorec G., Baley C., Sire O., Grohens Y. (2005). Characterization of interdiffusion between PVDF and stereoregular PMMA by using ATR–FTIR Spectroscopy. Macromol. Symp..

[24] Duckworth P., Richardson H., Carelli C., Keddie J. (2004). Infrared ellipsometry of interdiffusion in thin films of miscible polymers. Surf. Interface Anal..

[25] Ma W., Zhang J., Wang X., Wang S. (2007). Effect of PMMA on crystallization behavior and hydrophilicity of poly (vinylidene fluoride)/poly (methyl methacrylate) blend prepared in semi-dilute solutions. Appl. Surf. Sci..

[26] Rios L., Hidalgo M., Cavaille J., Guillot J., Guyot A., Pichot C. (1991). Polystyrene (1)/poly (butyl acrylate-methacrylic acid)(2) core–shell emulsion polymers. Part I. Synthesis and colloidal characterization. Colloid Polym. Sci..

[27] Gilbert R.G. (1995). Emulsion Polymerization: A Mechanistic Approach.

[28] Suresh K.I., Pakula T., Bartsch E. (2007). Synthesis Morphology and Rheological Behavior of Fluoropolymer-Polyacrylate Nanocomposites. Macromol. React. Eng..

[29] Yoshida H. (1997). Structure formation of PVDF/PMMA blends studied: Simultaneous DSC/FT–IR measurement. J. Thermal. Anal..

[30] Nishi T., Wang T. (1975). Melting point depression and kinetic effects of cooling on crystallization in poly (vinylidene fluoride)-poly (methyl methacrylate) mixtures. Macromolecules.

[31] Tashiro K., Kobayashi M., Tadokoro H. (1981). Vibrational spectra and disorder-order transition of poly (vinylidene fluoride) form III. Macromolecules.

[32] Bachmann M., Gordon W., Koenig J., Lando J. (1979). An infrared study of phase-III poly (vinylidene fluoride). J. Appl. Phys..

[33] Tashiro K., Takano K., Kobayashi M., Chatani Y., Tadokoro H. (1983). A preliminary X-ray study on ferroelectric phase transition of poly (vinylidene ruoride) crystal form I. Polym. Bull..

[34] Li Y., Tang S., Pan M.-W., Zhu L., Zhong G.-J., Li Z.-M. (2015). Polymorphic Extended-Chain and Folded-Chain Crystals in Poly (vinylidene fluoride) Achieved by Combination of High Pressure and Ion–Dipole Interaction. Macromolecules.

[35] Haris M.R., Kathiresan S., Mohan S. (2010). FT–IR and FT–Raman spectra and normal coordinate analysis of poly methyl methacrylate. Der Pharma Chemica.

[36] Peng Y., Wu P. (2004). A two dimensional infrared correlation spectroscopic study on the structure changes of PVDF during the melting process. Polymer.

[37] Mohammadi B., Yousefi A.A., Bellah S.M., Yousefi A.A., Bellah S.M. (2007). Effect of tensile strain rate and elongation on crystalline structure and piezoelectric properties of PVDF thin films. Polym. Test..

[38] Salimi A., Yousefi A. (2003). Analysis method: FTIR studies of β-phase crystal formation in stretched PVDF films. Polym. Test..

[39] Chen Y., Chen X., Zhou D., Shen Q.D., Hu W. (2016). Low-temperature crystallization of P(VDF–TrFE–CFE) studied by Flash DSC. Polymer.

[40] Shukla N., Thakur A.K. (2010). Nanocrystalline filler induced changes in electrical and stability properties of a polymer nanocomposite electrolyte based on amorphous matrix. J. Mater. Sci..

