# Supplementary material for: How Chain Intermixing Dictates the Polymorphism of PVDF in Poly(vinylidene fluoride)/Polymethylmethacrylate Binary System during Recrystallization: A Comparative Study on Core–Shell Particles and Latex Blend
Source: Polymers (Basel). 2017 Sep 14;9(9):448. doi: 10.3390/polym9090448 (PMC6418962; doi:10.3390/polym9090448)
Supplement: Supplementary file 1 [file polymers-09-00448-s001.pdf]

polymers

How Chain Intermixing Dictates the Polymorphism of PVDF in Poly(vinylidene fluoride)/ Polymethylmethacrylate Binary System during Recrystallization: A Comparative Study on Core/Shell Particles and Latex Blend

Yue Li [1,2], Guoqiang Zhang [3], Shaofeng Song [1], Haijun Xu [1], Mingwang Pan [1,*] and Gan-Ji Zhong [2,*]

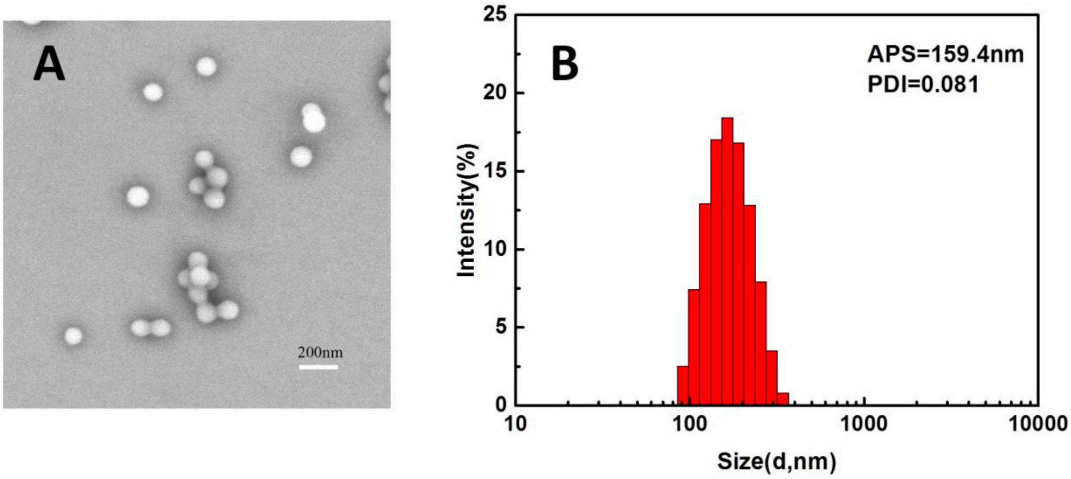

Figure S1 (A) SEM image and (B) size distribution of PMMA latex particles.

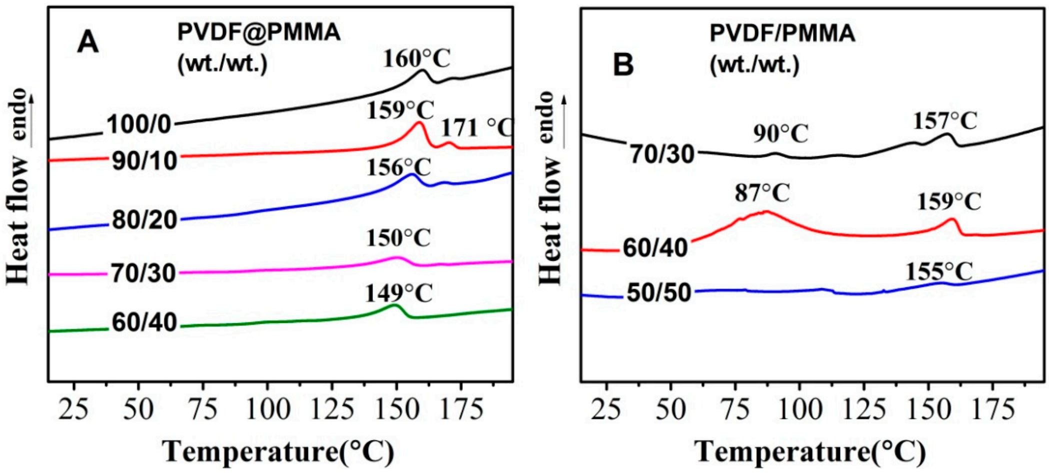

Figure S2. DSC first heating curves of (**A**) PVDF@PMMA, (**B**) PVDF/PMMA at various weight ratios of PVDF to PMMA. The cooling rate was −10 °C/min.

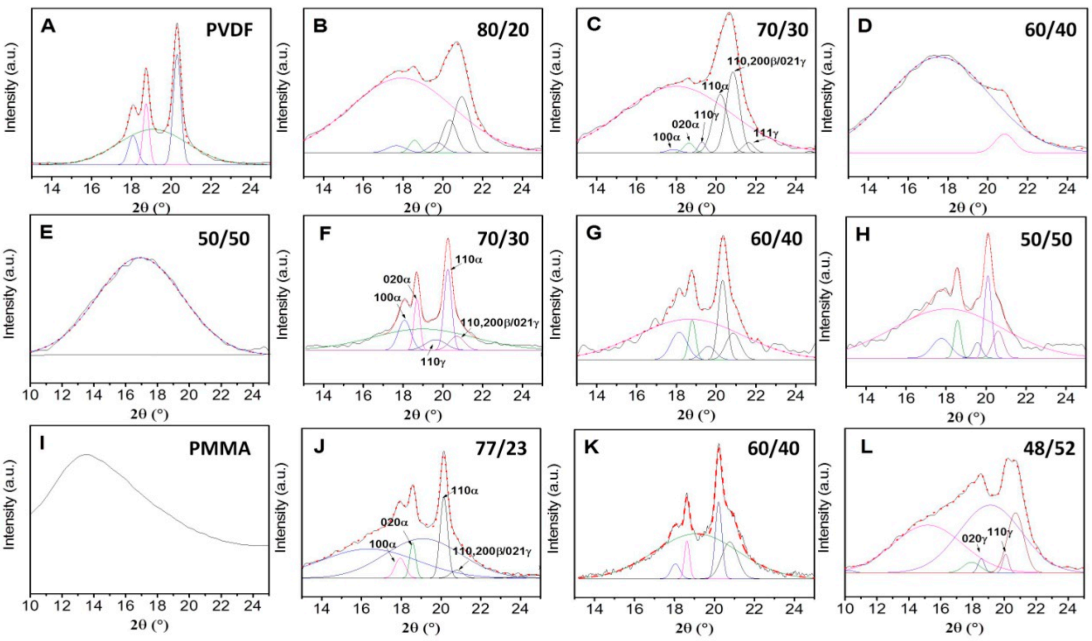

Figure S3. Deconvolution of the X-ray diffraction curves for (**A**) neat PVDF, (**I**) neat PMMA and three series of samples: (**B**–**E**) PVDF@PMMA, (**F**–**H**) PVDF/PMMA, (**J**-**L**) PVDF@c–PMMA.
